# Evolution of gene neighborhoods within reconciled phylogenies

**DOI:** 10.1093/bioinformatics/bts374

**Published:** 2012-09-03

**Authors:** Sèverine Bérard, Coralie Gallien, Bastien Boussau, Gergely J. Szöllősi, Vincent Daubin, Eric Tannier

**Affiliations:** ^1^Univ Montpellier2, UMR AMAP, Montpellier F-34000; ^2^LIRMM, CNRS, Univ Montpellier2, Montpellier F-34392, France; ^3^LBBE, UMR CNRS 5558, Université de Lyon 1, Villeurbanne F-69622, France; ^4^Department of Integrative Biology, UC Berkeley 4163A Valley Life Sciences Bldg Berkeley, CA 94720-3140, USA; ^5^INRIA Rhône-Alpes, Montbonnot F-38322, France

## Abstract

**Motivation:** Most models of genome evolution integrating gene duplications, losses and chromosomal rearrangements are computationally intract able, even when comparing only two genomes. This prevents large-scale studies that consider different types of genome structural variations.

**Results:** We define an ‘adjacency phylogenetic tree’ that describes the evolution of an adjacency, a neighborhood relation between two genes, by speciation, duplication or loss of one or both genes, and rearrangement. We describe an algorithm that, given a species tree and a set of gene trees where the leaves are connected by adjacencies, computes an adjacency forest that minimizes the number of gains and breakages of adjacencies (caused by rearrangements) and runs in polynomial time. We use this algorithm to reconstruct contiguous regions of mammalian and plant ancestral genomes in a few minutes for a dozen species and several thousand genes. We show that this method yields reduced conflict between ancestral adjacencies. We detect duplications involving several genes and compare the different modes of evolution between phyla and among lineages.

**Availability:** C++ implementation using BIO++ package, available upon request to Sèverine Bérard.

**Contact:** Severine.Berard@cirad.fr or Eric.Tannier@inria.fr

**Supplementary information:** Supplementary material is available at *Bioinformatics* online.

## 1 INTRODUCTION

A phylogenetic tree describes the kin relationships between a set of homologous objects. Non-homologous objects may have other types of relationships, such as interactions, functional relationships, co-expression or neighborhood between genes. Studying the pattern of descent of these relationships can be used to define homology between them, reconstruct ancestral relationships and build phylogenetic trees.

The evolution of gene proximity or interaction has been the subject of numerous recent studies. It is for example a way to assess co-evolution between genes, even if often co-evolution is detected by searching for similarities in gene trees, but without modeling explicitly the relation that make the genes co-evolve (Rodionov *et al.*, 2011; Tuller *et al.*, 2010).

Closer to our study, (Pinney *et al.*, 2007) and (Dutkowski and Tiuryn, 2009) or (Ma *et al.*, 2008) propose methods to reconstruct ancestral protein–protein interactions or gene neighborhoods based on a model of evolution allowing gene duplications. They, however, assume that the chronology of duplications is known, which often is not the case. (Patro *et al.*, 2011) define a general problem of network evolution without this assumption and give a heuristic solution for the comparison of two species. Our model considers the more specific problem of gene neighborhoods on chromosomes, but generalizes (Patro *et al.*, 2011)'s method in that it handles an arbitrary number of species and provides an exact solution to a less constrained problem.

Several methods are aimed at building ancestral chromosomes (which can be seen as relationships between genes). Most of these methods, however, ignore duplications and losses and are limited to gene families which have exactly one representative in each studied species (Alekseyev and Pevzner, 2009; Chauve and Tannier, 2008; Chauve *et al.*, 2010; Ma *et al.*, 2006; Ouangraoua *et al.*, 2011). The number of such gene families becomes smaller and smaller as the number of species grows. Some methods take as input gene trees allowing duplications and losses (Lajoie *et al.*, 2010; Muffato *et al.*, 2010) but do not model these events and treat them as noise that is removed for the construction of chromosomes by traveling salesman-like optimization methods. (Chauve *et al.*, 2010), (Ouangraoua *et al.*, 2011) or (Zheng and Sankoff, 2011) model duplications only in the context of whole genome duplications.

Here, we propose a method that takes a species tree and a set of gene trees as inputs, and models the gain and breakage of gene adjacencies along a pair of trees, taking duplications and losses into account. We consider two genes to be ‘adjacent’ if they are on the same chromosome in the same genome and no other gene is located between the two. We give an exact polynomial algorithm which minimizes the number of gains and breakages of adjacencies, or more generally, the gain/breakage cost of an evolutionary scenario for gene adjacencies. The result consists of sets of ‘adjacency trees’, which are phylogenetic trees describing the evolution of a family of homologous adjacencies (adjacencies that share a common ancestor and derived from it).

We assume that adjacencies evolve independently from each other, so we do not model the rearrangement explicitly (inversions, translocations etc.), but model their effect on adjacencies, which thus can undergo gains and breakages.

Doing this, we solve a problem that fits in the methodological program started by (Sankoff and El-Mabrouk, 2000), which mixes rearrangements and reconciliations of phylogenetic trees (a reconciliation is an annotation of gene tree nodes by duplication or speciation events, according to a species tree).

Algorithmically, the dynamic programming principle we use generalizes the Sankoff–Fitch (Fitch, 1971; Sankoff, 1975) parsimony algorithms on binary alphabets. Indeed, when there are no duplications nor losses in gene trees, adjacencies may be described by a binary character (presence or absence in a genome) evolving along the species tree, as in (Tang and Wang, 2005) or (Feijao and Meidanis, 2011). In our case, there is also a binary character (presence or absence of an adjacency), but evolving along pairs of reconciled gene trees.

The description of the method requires that we formally introduce the three kinds of trees we handle (species, gene and adjacency trees), as well as the definition of the optimization problem we propose (Section 2). We detail some properties of the solutions, pointing at the possible advantages and drawbacks of this approach in Section 3. Then in Section 4, we describe the algorithm which solves the problem. Proofs are provided in the Supplementary Material.

We implemented the algorithm and applied it to mammalian and plant genomes. We show that compared with other methods not explicitly modelling evolutionary events, we have more precise and less erroneous views at ancestral genome organization (Section 5). In addition, we are able to detect segmental duplications including several genes, and to visualize how much the modes of evolution are different according to the considered clades or lineages.

## 2 MODEL

All the trees in this article have one or more vertices, they are rooted and have maximum degree 3. A tree *T* induces a partial order on its nodes, where descendants are lower than ancestors.

For a tree *T*, *V* (*T*) denotes its vertex set and *L*(*T*) is leaf set (vertices with no descendants, *L*(*T*)⊆*V* (*T*)). For a node *N* of *T*, *T* (*N*) is the subtree of *T* rooted at *N*. *P*(*N*) is the parent of *N* (it is defined only if *N* is not the root). *L*(*N*) is the set of leaves of *T* (*N*).

We consider all trees to be annotated, which means here that each node *N* of a tree is labeled by

- A type of event *E*(*N*).
- A species *S*(*N*).

The events *E*(*N*) are taken from {*Extant*, *Spec*, *GDup*, *GLos*, *ADup*, *ALos*, *Break*}. These are abbreviations for ‘Extant’, ‘Speciation’, ‘Gene duplication’, ‘Gene loss’, ‘Adjacency duplication’, ‘Adjacency loss’ and ‘Adjacency breakage’. Together with the ‘Adjacency Gain’ (abbreviated *Gain*, which never labels the nodes of the trees as there is exactly one gain per adjacency tree), they are all the evolutionary events we consider.^1^ Note that *ALos* means the loss of an adjacency due to the concurrent loss of the two involved genes, while *Break* means the loss of an adjacency due to a rearrangement. In the objective function we only take *Gain* and *Break* into account, so both are given a cost *C*(*Gain*) and *C*(*Break*).

**Fig. 1.**
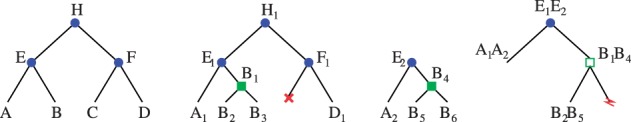
Examples of a species tree (left), two gene trees (middle) and an adjacency tree (right). Blue dots are speciation nodes. Leaves are extant (species, genes, adjacencies), except the one labeled by a red cross (gene loss) or a red flash (breakage). Green squares are (gene or adjacency) duplication nodes. Gene labels refer to the species they belong to. Every node of the adjacency tree is labeled by a couple of nodes from gene trees

All trees depend on a set of extant *genomes*, which are disjoint sets of genes plus binary relations on these sets of genes called ‘adjacencies’. The two genes of an adjacency are called its ‘extremities’. There are three types of trees (illustrated in Fig. 1), which have the following properties.

1. A species tree T_S_ describes the diversification of species. It is binary, and verifies *E*(*N*)= *Spec* for all internal node *N* and *E*(*N*)= *Extant* for all leaves *N*. All *S*(*N*) are distinct species and if *N* is an internal node, *S*(*N*) defines an ancestral species.
2. A gene tree T_G_ describes the evolution of a family of homologous genes along a species tree *T_S_*. All gene trees here are ‘reconciled’ with the ‘LCA (Last Common Ancestor) reconciliation’ (Goodman *et al.*, 1979) where all gene losses are represented by leaves, which means every node *N* verifies:
  - If *N* is a leaf, then *E*(*N*)∈{*Extant,GLos*}, and if *N* is an internal node, then *E*(*N*)∈{*Spec,GDup*}.
  - If *E*(*N*)=*Extant*, then there is a gene *G*(*N*) that belongs to *S*(*N*) and all such genes are distinct.
  - If *E*(*N*)= *GDup* then the children *N*1 and *N*2 of *N* are such that *S*(*N* 1)= *S*(*N*2)= *S*(*N*)
  - If *E*(*N*)= *Spec* then the children *N*1 and *N*2 of *N* are such that there are two edges *AA*1 and *AA*2 of *T_S_* such that *P*(*A*1)= *P*(*A*2)= *A* and *S*(*N*)= *S*(*A*), *S*(*A*1)= *S*(*N*1), and *S*(*A*2)= *S*(*N*2).
  - Let 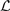 be the set of leaves of *T_G_*(*N*); Let 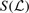 be the set of all extant species which are descendants of some *S*(*l*), 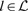; Let now *N_S_* be the lowest node in *T_S_* such that 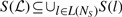; Then, *S* (*N*)=*S*(*N_S_*).
3. An adjacency tree T_A_ describes the descent pattern of adjacencies. As adjacencies are pairs of genes, they follow the evolution of genes: if an adjacency *AB* descents from an adjacency *CD*, then *A* descents from *C* and *B* from *D*. So adjacency trees are defined given a set of reconciliated gene trees 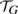 and have to follow their LCA reconciliations. Formally, every node *N* of an adjacency tree verifies
  - If *N* is a leaf, then *E*(*N*)∈{*Extant,GLos,ALos,Break*} and if *N* is an internal node, then *E*(*N*)∈{*Spec,GDup,ADup*}
  - If *E*(*N*)≠ *Break*, then there is a couple *A*(*N*)= *XY* of gene tree nodes *X* and *Y* (possibly from two different gene trees) such that *S*(*N*)= *S*(*X*)= *S*(*Y*).
  - If *E*(*N*)= *Extant*, then *G*(*X*)*G*(*Y*) is an adjacency.
  - If *E*(*N*)= *GLos*, then *E*(*X*)= *GLos* or *E*(*Y*)= *GLos* (and not both).
  - If *E*(*N*)= *ALos*, then *E*(*X*)= *E*(*Y*)= *GLos*.
  - If *E*(*N*)= *Spec*, then *E*(*X*)= *E*(*Y*)= *E*(*N*). In addition, *N* has two children *N*1 and *N*2 and either *E*(*N*1)= *Break* (respectively, *E*(*N*2)= *Break*)) or *A*(*N*1) (respectively, *A*(*N*2)) is a couple of children of *X* and *Y* .
  - If *E*(*N*)= *ADup*, then *E*(*X*)= *E*(*Y*)= *GDup*. In addition, *N* has two children *N*1 and *N*2 either *E*(*N*1)= *Break* (respectively, *E*(*N*2)= *Break*)) or *A*(*N*1) (respectively, *A*(*N*2)) is a couple of children of *X* and *Y* .
  - If *E*(*N*)= *GDup*, then *E*(*X*)= *GDup* or *E*(*Y*)= *GDup* (suppose it is *Y*). In addition, *N* has only one child *N*1 and either *E*(*N*1)= *Break* or *A*(*N*1) is a couple of genes composed of *X* and one child of *Y* .

An ‘adjacency forest’ is a set of adjacency trees, such that for two nodes *N*1 and *N*2 in this forest, *A*(*N*1)≠ *A*(*N*2), and such that for each adjacency *A* from any species, there exists a leaf *L* in the forest, which verifies *A*(*L*)= *A*.

The cost of an adjacency tree *T_A_*, is

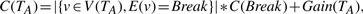

where *Gain*(*T_A_*) is computed in this way: if the root *R* of *T_A_* is such that *A*(*R*)= *XY* and either

- *P*(*X*)= *P*(*Y*) or
- *X* is the root of a gene tree, and either *Y* is also a root, or *S*(*P*(*Y*))≠ *S*(*Y*)

then *Gain*(*T_A_*) = 0, else *Gain*(*T_A_*) = *C*(*Gain*). The cases where *Gain*(*T_A_*) = 0 are those arising from tandem duplications or those where the adjacency can have been gained earlier in the evolution.

The cost of an adjacency forest is the sum of the costs of all adjacency trees.

The problem we address is to take as input a species tree, a set of gene trees and a set of extant adjacencies, and to compute an adjacency forest of minimum cost. We give a polynomial algorithm which gives one optimal solution.

## 3 PROPERTIES

### 3.1 The cost of a duplication or loss event

The optimization focuses only on breaks and gains of adjacencies. The dynamic programming technique we use does not allow to count duplication and loss events in the objective function. This is because we make the hypothesis of independent evolution of couples of genes, and as long as one gene has its own events and belongs to several couples, this independence is broken.

Nevertheless, duplication events have an importance for the solutions. The duplication of an adjacency has the same cost as the independent duplication of two genes, but the events can still be discriminated because the two do not have the same effect: the independent duplications propagate only one adjacency, and the joint duplication propagates two. It is thus possible to catch the places where a joint duplication is advantageous in terms of gains and breaks.

### 3.2 The linearity of genomes

In extant genomes, one gene can participate in at most two adjacencies. We have not required this property in the input of the program because it is not used, and in this way we could easily adapt the problem to other kinds of relationships. The drawback of this is that there is no need that in ancestral genomes, genes participate to at most two adjacencies.

(Feijao and Meidanis, 2011) prove that in a duplication-free framework, where Fitch's algorithm is applied on the presence and absence of adjacencies, choosing the absence whenever there is a choice to make ensures that the resulting genomes are linear. When there are duplications, it is not necessarily the case and it can be seen in the data, where some conflicts remain. But as we will see in the last section, the amount of conflict is reduced compared with other kinds of algorithms, and can be used to assess the quality of the gene trees, as well as the quality of the model.

### 3.3 The chronology of duplications

No chronology of duplications is required in the input as in (Pinney *et al.*, 2007), (Dutkowski and Tiuryn, 2009) or (Ma *et al.*, 2008). But a chronology can be derived from the output. Indeed an adjacency duplication means that two genes are duplicated together, while two nodes of an adjacency tree such that one is the descendant of the other and both are gene duplication events define a directed relation between the two duplications, even if they are not comparable from the gene trees (not in the same tree or not comparable in one tree). But this relation is not necessarily an order relation. There are examples where temporal relationships defined by adjacency trees contradict the partial order of the nodes of one gene tree: see such an example in Figure S1 in Supplementary Material. (Patro *et al.*, 2011) proscribe this kind of conflict and propose a heuristic principle to get rid of it when it happens.

### 3.4 Tandem duplications

Tandem duplications are special types of duplications, where the two duplicates are adjacent. Here, tandem duplications are not modeled explicitly as a different event from ordinary duplications. However, tandem duplications of one gene are indirectly taken into account: they cost zero (as the gain of an adjacency between two children of a duplication node is costless; see Section 2), while a non-tandem duplication of one gene can cost one breakage plus two gains when one duplicate is inserted between two other genes.

### 3.5 The orientations of the genes

It is possible to take the orientation of the genes into account by duplicating each gene into two gene extremities and define adjacencies as relations between gene extremities instead of genes (the extremities of an adjacency are gene extremities in that case). The current implementation can be used in this way, it is just a matter of formatting the input. In this case, one gene extremity is supposed to participate in only one adjacency, and tandem duplications are not handled anymore, because there are no duplications of only one gene extremity.

## 4 ALGORITHM

### 4.1 Restriction to two gene trees

We first restrict the problem to the comparison of two gene trees, without loss of generality. To do this, the extant adjacencies are clustered according to the following relation between two distinct adjacencies *AB* and *CD*:

1. *A* and *C* are in the same gene tree, noted *G*1, as well as *B* and *D*, in a tree noted *G*2 (the roles of *A* and *B* and of *C* and *D* may be exchanged).
2. if *G*1≠ *G*2, then there are two nodes *N*1∈*G*1,*N*2∈*G*2 such that *S*(*N*1)= *S*(*N*2) and *A* and *C* are descendants of *N*1, while *B* and *D* are descendants of *N*2.
3. if *G*1= *G*2, then the lowest common ancestor of *A* and *B* is the same node as the lowest common ancestor of *C* and *D* (it is necessarily a duplication node).

This relation between adjacencies satisfying all conditions is an equivalence relation (reflexive, symmetric and transitive). Equivalence classes are treated independently. This is justified by the following lemma, whose proof stands in the Supplementary Material.

Lemma 1. *If there is a tree of adjacencies which contains adjacencies AB and CD, then AB and CD are in the same equivalence class.*

In other words, if *AB* and *CD* are not in the same class they cannot be homologous. The converse is not true however. Solutions for one class may consist of several adjacency trees.

This clustering allows to divide the problem into equivalence classes, which concern one or two gene trees. If in an equivalence class, adjacencies have extremities in the same tree, by definition of the classes, there is a common ancestor to all pairs of extremities of adjacencies. By removing this vertex, we get two trees rooted at its children, and all adjacencies have one extremity in each of these two trees.

So we may restrict ourselves to this case where we have exactly two gene trees and all adjacencies are between these two trees. Moreover, we may suppose that each tree is rooted at the lowest common ancestor of all genes involved in adjacencies of the chosen class, because we may simply consider the subtree rooted at this vertex. This yields that the two roots are necessarily assigned to the same species.

## 4.2 Recurrence formulas

Formally, we have two gene trees *T_G_*^1^ and *T_G_*^2^, extant adjacencies have one extremity in each tree, and if *R*^1^ and *R*^2^ are the respective roots of *T_G_*^1^ and *T_G_*^2^, then *S*(*R*^1^) = *S*(*R*^2^).

For a pair of nodes (*v*^1^,*v*^2^)∈*V* (*T_G_*^1^) × *V*(*T_G_*^2^) such that *S*(*v*^1^)*=S*(*v*^2^), we compute two values, *c*_1_(*v*^1^,*v*^2^) and *c*_0_(*v*^1^,*v*^2^) by recurrence formulas described in the sequel. Remark that we only consider pairs of nodes annotated with the same species because an adjacency is always linking genes from the same genome. We prove that these numbers have the following properties (proofs are in the Supplementary Material, Appendix 2).

Theorem 1.

- *c*_1_(*v*^1^,*v*^2^) *is the minimum cost of an adjacency forest F for the adjacencies between two gene trees T_G_*^1^*(v*^1^*) and T_G_*^2^*(v*^2^*)*, *such that there is a node N in F with A*(*N*)= *v*^1^*v*^2^.
- *c*_0_(*v*^1^,*v*^2^) *is the minimum cost of an adjacency forest F for the adjacencies between two gene trees T_G_*^1^*(v*^1^*) and T_G_*^2^*(v*^2^*)*, *such that there is no node N in F with A*(*N*)= *v*^1^*v*^2^.

In consequence, the minimum cost of an adjacency forest will be given by computing the minimum between *c*_1_(*R*^1^,*R*^2^) and *c*_0_(*R*^1^,*R*^2^).

The recurrence for the computation of *c*_1_(*v*^1^,*v*^2^) and *c*_0_(*v*^1^,*v*^2^) follows a case analysis, according to the type of event associated to *v*^1^ and *v*^2^. The roles of *v*^1^ and *v*^2^ are symmetrical. We note *ca*(*v*) and *cb*(*v*) the two children of a node *v*.

**Case 1.** *E*(*v*^1^)= *Extant* and *E*(*v*^2^)= *Extant*.

If *v*^1^*v*^2^ is an adjacency then *c*_1_(*v*^1^,*v*^2^)=0 and *c*_0_(*v*^1^,*v*^2^)=∞; else *c*_1_(*v*^1^,*v*^2^)=∞ and *c*_0_(*v*^1^,*v*^2^)=0.

**Case 2.** *E*(*v*^1^)= *GLos* and *E*(*v*^2^)≠ *GLos*.

In this case *c*_1_(*v*^1^,*v*^2^)=0 and *c*_0_(*v*^1^,*v*^2^)=0.

**Case 3.** *E*(*v*^1^)= *GLos* and *E*(*v*^2^)= *GLos*.

In this case *c*_1_(*v*^1^,*v*^2^)=0 and *c*_0_(*v*^1^,*v*^2^)=0. This case has to be distinguished from the previous one for the backtracking procedure described in the following subsection.

**Case 4.** *E*(*v*^1^)∈{*Extant*,*Spec*} and *E*(*v*^2^)= *GDup*.


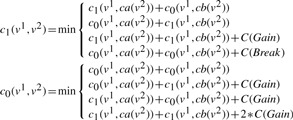


**Case 5.** *E*(*v*^1^)= *Spec* and *E*(*v*^2^)= *Spec*.

Suppose without loss of generality that *S*(*ca*(*v*^1^))=*S*(*ca*(*v*^2^)) and *S*(*cb*(*v*^1^))=*S*(*cb*(*v^2^*)).


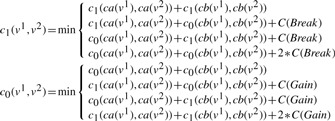


**Case 6.** *E*(*v*^1^)= *GDup* and *E*(*v*^2^)= *GDup*.

In this case *c*_1_ (*v*^1^,*v*^2^)= *min*(*D*1,*D*2,*D*12) where *D*1 is the cost in the case the *v*^1^ duplication comes first, *D*2 is the cost in the case the *v*^2^ duplication comes first, *D*12 is the cost in the case where the *v*^1^ and *v*^2^ duplications are simultaneous.

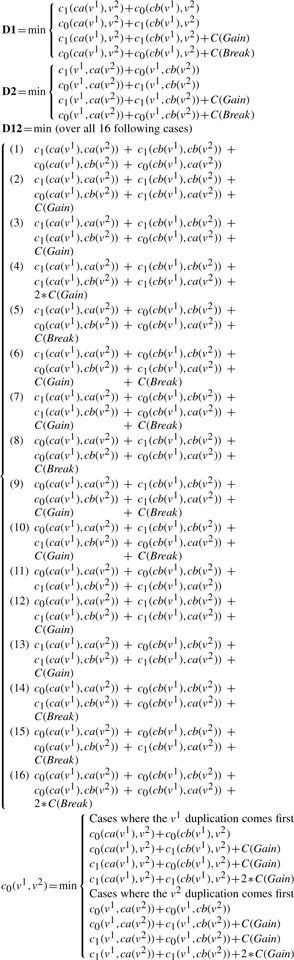



We do not examine the case *E*(*v*^1^)= *Extant* and *E*(*v*^2^)= *Spec* because in this case *S*(*v*^1^)≠ *S*(*v*^2^).

The algorithm implements these recurrence formulas in an iterative way following a dynamic programming technique, by computing the costs in a post-order traversal of the couples of tree nodes.

## 4.3 Backtracking

The recurrence formulas allow the construction of a cost matrix which rows are the nodes of the first gene tree, and columns are the nodes of the second gene tree. The nodes of the adjacency forest are constructed while backtracking on the cost matrix starting at min(*c*_1_(*R*^1^,*R*^2^),*c*_0_(*R*^1^,*R*^2^)). The backtracking procedure classically follows each cost on the chosen line in the recurrence formulas, creating adjacency trees from root to leaves. A node *N* with *A*(*N*)= *v*^1^*v*^2^ is created each time *c*_1_(*v*^1^,*v*^2^) is chosen. The event labeling this node depends on the events labeling *v*^1^ and *v*^2^ : *Extant* for Case 1, *GLos* for Case 2, *ALos* for Case 3, *GDup* for Cases 4 and 6. (D1&D2), *Spec* for Case 5 and *ADup* for Case 6 (D12). A node *N* with *E*(*N*)= *Break* is created each time there is a *C*(*Break*) in the chosen formula.

Edges between the nodes follow the pattern of descent between adjacencies:

- *Break* nodes are leaves, and their parent are the nodes constructed in the formula where *C*(*Break*) occurs;
- In Cases 4 and 6 (D1&D2), there is an edge between *v*^1^*v*^2^ and one of *v*^1^*ca*(*v*^2^), *v*^1^*cb*(*v*^2^), *ca*(*v*^1^)*v*^2^, *cb*(*v*^1^)*v*^2^, if *c*_1_ is chosen for either of them.
- In Cases 5 and 6 (D12), there is an edge between *v*^1^*v*^2^ and one or two of *ca*(*v*^1^)*ca*(*v*^2^), *cb*(*v*^1^)*cb*(*v*^2^), *cb*(*v*^1^)*ca*(*v*^2^), *ca*(*v*^1^)*cb*(*v*^2^) if *c*_1_ is chosen for either of them (there can be arbitrary choices for equivalent solutions).

Recurrence formulas imply that the backtracking procedure does not create twice the same node: each formula computes the cost for *v*^1^*v*^2^ between pairs of nodes where at least one is a descendant of *v*^1^ or *v*^2^.

**Fig. 2.**
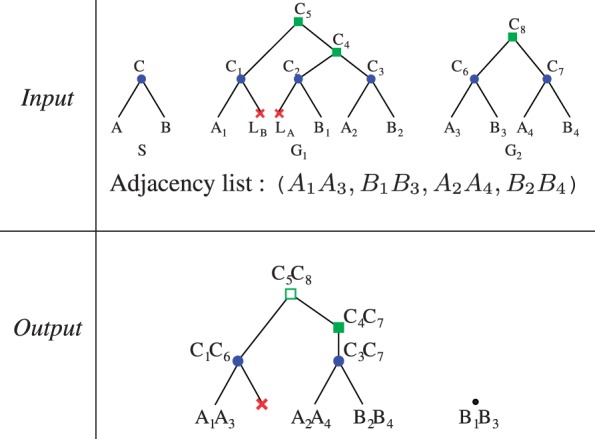
Example of the application of the algorithm on two genes trees, *G*_1_ and *G*_2_, a species tree *S* and an adjacency list shown on the line *Input*. The costs are *C*(*Gain*)= *C*(*Break*)= 1. All the costs *c_b_*(*E_i_*,*E_j_*) are computed for *b*∈0,1, *E* ∈*A*,*B*,*C*, *i*,*j* ∈[1..8], with *E_i_* in *G*_1_ and *E_j_* in *G*_2_. As a result *c*_0_(*C*_5_,*C*_8_) = 2 while *c*_1_(*C*_5_,*C*_8_) = 1. Therefore, the adjacency forest on the line *Output* contains *C*_5_*C*_8_. The left tree has cost 0 while the right one costs *C*(*Gain*)= 1 for the gain of the adjacency *B*_1_*B*_3_

An example of an algorithm input and output is drawn on Figure 2.

## 4.4 Complexity

The algorithm takes as input a dataset composed by a species tree, several gene trees and a list of adjacencies. It first computes the equivalence classes of adjacencies. Then for each class it constructs two subtrees to compute *c*_0_ and *c*_1_ costs on their roots and applies the backtracking procedure. The algorithm outputs the adjacency forest resulting from the union of all adjacency forests built on each class.

Let *n* be the number of gene trees and *k* be the maximum size of a tree. The algorithm runs in *O*(*n*^2^ ×*k*^2^). Indeed, the maximum number of adjacency equivalence classes is bounded by *O*(*n*^2^), while for each equivalence class, every couple of node is examined with a constant-time case analysis.

In practice, the number of equivalence classes is much closer to *n* than to *n*^2^ and trees are small compared with the total number of genes. For all datasets we tested, including dozens of species and thousand of genes, the execution time was under 10 min.

## 5 IMPLEMENTATION AND APPLICATION

We implemented the algorithm using the Bio++ platform (Dutheil *et al.*, 2006). The program, named *DeCo* (Detection of Co-evolution or DeCoration of trees), takes as input a species tree, a set of genes along with the species they are in, a set of adjacencies and a set of gene trees.

We tested it on four datasets, with costs *C*(*Gain*)= *C*(*Break*)= 1. The first and second datasets are based on 5039 gene trees from the Ensembl database (release 57) restricted to mammalian assembled genomes (11 species).^2^ The first set of trees are those provided in this database, made according to the TreeBeST pipeline (Vilella *et al.*, 2009). The second consists of the trees reconstructed by the PhylDog method (Boussau *et al.*, 2012), with an explicit model of duplication and losses of trees. Both sets of trees were reconciled according to the LCA method (Goodman *et al.*, 1979), which gives gene trees with the properties written in Section 2).

Then, we computed ancestral adjacencies according to the method described here, and compared with the ‘pairwise alternative’, an implementation of the principles used by (Chauve and Tannier, 2008), (Muffato *et al.*, 2010), (Bertrand *et al.*, 2010) or (Boussau *et al.*, 2012), in which adjacencies are constructed by comparing couples of species (the method is described in the Supplementary Material, Appendix 3) instead of all genomes together.

**Fig. 3.**
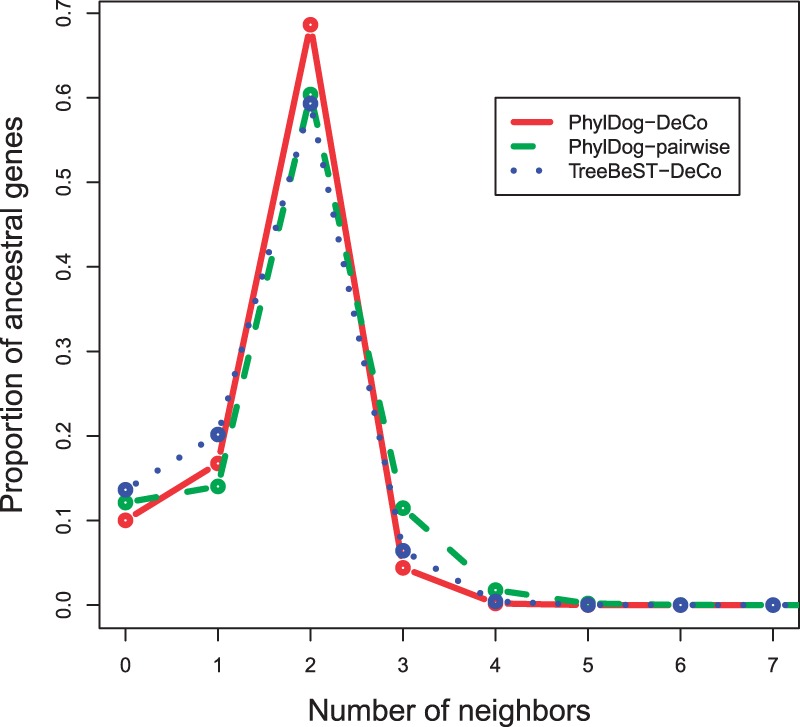
Proportion of genes having *k* neighbors, function of *k*. Red plain line is obtained with DeCo and PhylDog trees. Green dashed line is obtained with PhylDog trees and the pairwise alternative. Blue dotted line is obtained with TreeBeST trees and DeCo

We computed the degree of each ancestral gene, that is, the number of adjacencies which has it as an extremity. As shown in Figure 3, most ancestral genes have degree 2, which means the signal of linearity of the ancestral genomes is recovered. We can observe the gain obtained by using PhylDog trees instead of TreeBeST trees (red plain versus blue dotted line), and the gain obtained by using DeCo instead of the pairwise alternative (red plain versus green dashed line). These two gains are nearly equivalent, showing that to get better ancestral genomes, we need good trees as well as good adjacency inference algorithms. Better trees tend to give a better estimate of the ancestral gene content, minimizing the degree 0 (probably wrong) genes, while the adjacency inference algorithm may minimize the number of genes with degree *>*2: convergent evolution can yield false ancestral adjacencies, which add to the two true ones. Convergent evolution is impossible to handle in a pairwise method.

**Fig. 4.**
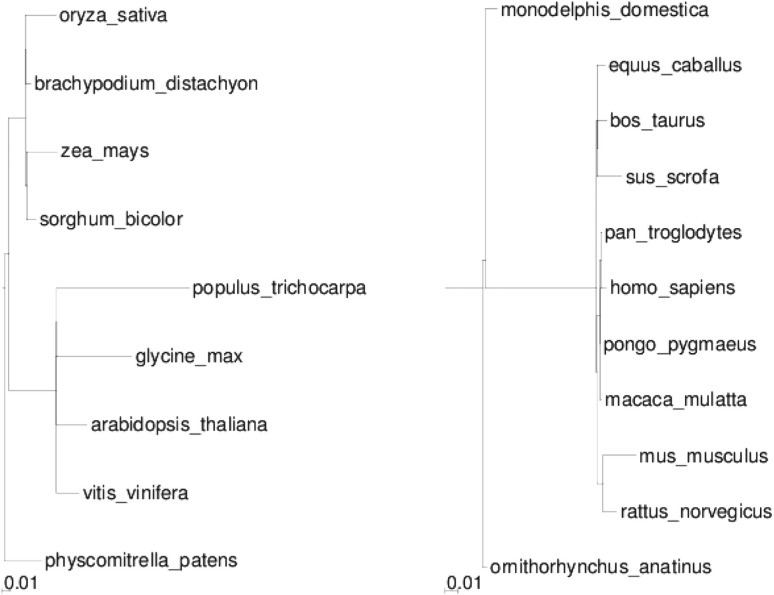
Angiosperm and mammalian phylogenies, where branch lengths are proportional to the number of adjacency duplications normalized by the number of genes. The scale is indicated at the bottom left of the two figures

The third and fourth datasets are constructed from the Ensembl (release 65) and EnsemblPlant (release 12) databases, restricted to some assembled mammalian (11 species, 19 217 gene trees with an average of 10 genes) and angiosperm (9 genomes, 35 182 gene trees, with an average of 9 genes) species. We chose these two clades for a phylogenetic comparison because the estiated divergence times are similar, and there are approximatively as many assembled genomes in both. We compared the number of segmental duplications involving more than one gene in these two datasets. In Figure 4, phylogenetic trees of mammals and angiosperms are drawn, in which branch length is the number of pairs of genes duplicated together over the total number of ancestral genes found in the same branch. We find that on average branch lengths are more than three times longer in plants, indicating genome architectures rapidly evolving compared with slow mammalian ones. Angiosperm genomes have been shaped by several whole genome duplications: at the basis of monocots, a triplication at the basis of dicots, plus one event on the Maize and Poplar lineages, and two on the Arabidopsis one. These events probably create a long branch in Poplar, or Glycine, but are not always visible (*e.g.* in *Arabidopsis*) due to differentiated losses which blurred the synteny signal. The difference in branch length can partly be due to whole genome duplications. But measuring the average size of the duplicated segments by computing

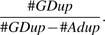

we found no significant difference between the two phyla (= 1.08 on average among all branches for both), indicating that the changes in genome architectures following a whole genome duplication are not fully accessible to this method. The long branch at the basis of eutheria would deserve more studies to know to which extend it is artifactual and due to the quality of gene trees.

## 6 PERSPECTIVES

The algorithm can easily be extended to handle other relations than adjacencies (interactions, regulations, co-expression or any functional relation which can evolve by gain or breakage like adjacencies). It can be seen as even more adapted to less constrained relations (without a linear organization). Indeed, if a gene is lost, no adjacency is automatically and freely gained between its two neighbors in this model. But the computation time should be higher for other relations, as the possible number of relations is a quadratic function of the number of genes, while the number of adjacencies only grows linearly.

Possible extensions can be to include transfers (Doyon *et al.*, 2011), incomplete lineage sorting or gene conversion (Rasmussen and Kellis, 2012) to the possible events. And also to allow other types of reconciliations than the LCA one. More flexible cost functions for duplications may also be desirable, but in this case the independent evolution between adjacencies is lost, and the use of dynamic programming does not seem generalizable.

## Footnotes

^1^Even if ‘Extant’ is not an evolutionary event, it is included because it annotates some tree leaves.
